# Prevalence of depressive and anxiety symptoms among patients consulting a rare disease outpatient clinic for rare and undiagnosed diseases in China

**DOI:** 10.1186/s13023-026-04359-6

**Published:** 2026-04-29

**Authors:** Xin Huang, Xin Liu, Xuelin Yang, Rong Chen, Chenyang Pei, Jingyuan Zhang, Min Shen, Shuyang Zhang

**Affiliations:** 1 Department of Rare Diseases, Peking Union Medical College Hospital (PUMCH), Chinese Academy of Medical Sciences & Peking Union Medical College; State Key Laboratory of Complex Severe and Rare Diseases, PUMCH; Department of Rheumatology and Clinical Immunology, PUMCH; National Clinical Research Center for Dermatologic and Immunologic Diseases (NCRC-DID), Ministry of Science & Technology; Key Laboratory of Rheumatology and Clinical Immunology, Ministry of Education, Beijing, China; 2 School of Population Medicine and Public Health, Chinese Academy of Medical Sciences & Peking Union Medical College, Beijing, China; 3 MRC Centre for Global Infectious Disease Analysis, Jameel Institute, School of Public Health, Imperial College London, London, UK; 4 Institute of Medical Information and Library, Chinese Academy of Medical Sciences & Peking Union Medical College, Beijing, China; 5 School of Health Policy and Management, Chinese Academy of Medical Sciences & Peking Union Medical College, Beijing, China; 6 Department of Rare Diseases, Peking Union Medical College Hospital (PUMCH), State Key Laboratory of Complex Severe and Rare Diseases, Chinese Academy of Medical Sciences & Peking Union Medical College, PUMCH, Beijing, China; 7 Department of Cardiology, Peking Union Medical College Hospital (PUMCH), Chinese Academy of Medical Sciences & Peking Union Medical College, Beijing, China

## Abstract

**Background:**

Patients with rare diseases often experience significant psychological distress. This study aims to assess the prevalence of depressive and anxiety symptoms and identify associated factors among patients consulting a rare disease outpatient clinic for rare and undiagnosed diseases in China.

**Methods:**

A clinic-based cross-sectional study was conducted from February to June 2024, with 201 participants recruited. Sociodemographic, health-related characteristics, and psychological symptoms were assessed using validated instruments. Multivariable logistic regression was performed to identify factors associated with depressive and anxiety symptoms.

**Results:**

Overall, 68.2% of participants reported depressive symptoms, and 41.3% reported anxiety symptoms. Poor sleep quality (OR = 10.298, 95%CI: 1.915–55.367) and pain or discomfort (OR = 2.292, 95% CI: 1.143–4.596) were associated with a higher likelihood of depressive symptoms. For anxiety symptoms, confrontation (OR = 1.143, 95% CI: 1.042–1.253) and acceptance-resignation (aOR = 1.166, 95% CI: 1.051–1.294) were positively associated with anxiety symptoms. Higher social support was a protective factor of both depressive symptoms (aOR = 0.893, 95%CI: 0.813–0.98) and anxiety symptoms (aOR = 0.885, 95%CI: 0.817–0.959).

**Conclusions:**

These findings highlight the substantial psychological burden faced by patients consulting a rare disease outpatient clinic in China and underscore the importance of integrating mental health support into the multidisciplinary care. Routine psychological screening and timely support can improve patients’ overall well-being and quality of life.

## Introduction

Rare diseases, although each affecting fewer than 1 in 2,000 individuals, collectively affect approximately 300 million people worldwide. Patients with rare diseases, particulary those in low- and middle-income countries, often experience substantial marginalization and inadequate access to timely diagnosis and care [1]. In China, an estimated 16.8 million individuals, including 11.8 million children, are currently living with rare diseases, placing a significant burden on the healthcare system and society [2]. Rare diseases encompass a wide range of conditions with significant heterogeneity, yet delayed diagnosis remains a common challenge faced by many rare diseases patients [3]. Given the diversity and complexity of rare diseases, there is a growing recognition of the need for specialized interdisciplinary approaches to diagnosis and clinical care [4]. In this context, Peking Union Medical College Hospital (PUMCH) launched a joint outpatient clinic for rare diseases on April 10, 2023, to provide multidisciplinary and collaborative care for patient with rare and undiagnosed diseases.

Patients with rare diseases often face multiple challenges, including limited access to disease-related information, restricted treatment and management options, and financial burden, all of which contribute to psychological distress. Previous research has shown that depression, anxiety and other emotional issues are highly prevalent among adults with rare diseases [5]. Another study reported that 90% of rare disease patients experience mental health challenges, with over 30% having contemplated suicide [6]. The diagnosis of rare diseases not only has a profound impact on the mental health of affected individuals but also poses significant challenges to the psychological well-being of their entire families [7]. However, the psychological needs of patients with rare disease are often insufficiently addressed in clinical care [6]. In China, evidence on the mental health status of patients with rare disease remains limited, and more systematic research is needed.

Therefore, this study aimed to investigate the mental health status of patients attending comprehensive rare disease outpatient clinics in China. Through this research, we seek to provide valuable insights into the psychological impact of rare diseases and the support required for patients in the Chinese context.

## Methods

### Study design and participants

This cross-sectional study was conducted at the joint outpatient clinic for rare diseases at PUMCH, from February 1, 2024, to June 30, 2024. Participants were recruited during their outpatient visits at the clinic. Each participant was approached and invited to participate by trained medical professionals, who explained the study’s purpose and procedures. All participants were provided with written informed consent forms, detailing the scope of the study, potential risks, and benefits. The inclusion criteria were as follows: (1) participants are patients with rare diseases or those presenting with rare disease symptoms; (2) able to understand the questions posed by the researcher; (3) voluntarily signed the informed consent. Due to the real-world outpatient setting where many patients are actively navigating their diagnostic evaluations, participants were enrolled and analyzed as a collective cohort based on self-reported status and clinical presentation.

#### Sample size calculation

The sample size was calculated using the formula: N = [Z_(α/2)_^2^ ×*p*×(1-*p*)]/d^2^. Based on previous research [8], the prevalence of depressive symptoms and anxiety symptoms among patients consulting a center for rare and undiagnosed diseases was 40.7% and 27.5%, respectively. With a significance level of α = 0.05, a margin of error of d = 0.1, and an effective response rate of 80%, the minimum required sample size was 120 participants.

### Measurement

#### Sociodemographic

Sociodemographic characteristics including age, gender, marital status, residence, educational level, household annual income, were collected. Health related information including history of chronic diseases, sleep quality, and whether did they experienced pain or discomfort, and the walking, self-care problems, was collected. Information about rare disease related variables was collected, including whether participants had been diagnosed with a rare disease, had been misdiagnosed with other diseases, had undergone genetic testing, had received multidisciplinary team (MDT) services during treatment, had been hospitalized, and the number of hospitals they had visited.

#### Depressive symptoms

The nine-items Patient Health Questionnaire (PHQ-9) was used to assess the severity of individual depressive symptoms in the past two weeks. This scale was developed by Spitzer and has been validated among patients. Each item is rated on a four-point Likert scale with a range from 0 to 3. The total score ranges from 0 to 27, with 0–4 indicating no depressive symptoms, 5–9 representing mild symptoms, 10–14 moderate symptoms, and 15 or above indicating severe symptoms. The Cronbach’s alpha of the PHQ-9 in this study was 0.862 [9, 10].

#### Anxiety symptoms

The seven-item Generalized Anxiety Disorder Scale (GAD-7) is a quantitative assessment tool recommended by the American Psychiatric Association and it was used to measure the severity of generalized anxiety symptoms over the past two weeks. Its reliability and validity have been well-established in domestic studies [11]. The items are rated on a four-point Likert scale with a range from 0 to 3. The total score ranges from 0 to 21, with 0–5 indicating no anxiety symptom, 6–9 mild symptom, 10–14 moderate symptom, and 15–21 severe symptom. In this study, the Cronbach’s alpha for the GAD-7 was 0.918.

#### Perceived social support

The Perceived Social Support Scale (PSSS) was developed by Li, et al. in 2017 and has been used among the Chinese population [12]. This scale measures the level of perceived social support among respondents during the past 2 weeks. It contains two items, and each item was scored from 0 to 10 (0 = strongly disagree; 10 = strongly agree), and the overall score is calculated by summing the scores of the two items. A higher score indicates a higher level of perceived social support. In this study, the Cronbach’s alpha of this scale was 0.616.

#### Self-perceived burden

The Self-Perceived Burden Scale (SPBS), developed by Cousineau et al., which is used to assess an individual’s subjective burden [13]. The scale consisted of 10 items and comprises three dimensions, including emotional burden, economic burden, and physical burden. It employs a five-point Likert scale, where 1 indicates “never” and 5 indicates “always”. The total score ranges from 10 to 50, with higher scores indicating a more severe self-perceived burden (SPB). Scores below 20 suggest no significant SPB, 20–29 indicate mild SPB, 30–39 moderate SPB, and 40 or above severe SPB. The scale has been translated and validated in China and has demonstrated good reliability and validity in cancer patient populations [14]. In this study, the Cronbach’s alpha for the SPBS was 0.947.

#### Coping style

The Medical Coping Modes Questionnaire (MCMQ) was developed by Hermanan F and is one of the scales used to measure coping strategies in patients [15]. A revised Chinese version of the questionnaire was adapted and has been validated in Chinese populations with chronic diseases, demonstrating good reliability and validity [16]. This version includes 20 items with three dimensions: “Confrontation,” “Avoidance,” and " acceptance-resignation.” Each item is scored on a scale of 1 to 4, with eight items being reverse scored. Higher average scores in a specific dimension indicate a greater tendency to adopt that coping strategy when dealing with illness. In this study, the Cronbach’s alpha for the MCMQ was 0.652.

### Statistical analysis

Continuous variables, such as age, were presented as means and standard deviations (Mean ± SD), while categorical variables, such as gender, marital status, and education level, were expressed as frequencies (n) and percentages (%). The Chi-square test was used to compare differences between two groups for categorical data, while the t-test was used for continuous data. To identify factors associated with depressive and anxiety symptoms, multivariable logistic regression analysis was conducted. This analysis accounted for multiple potential covariates, including sleep quality, pain or discomfort, social support, and coping styles. This study adhered to the Strengthening the Reporting of Observational studies in Epidemiology (STROBE) criteria and all statistical analyses were performed using SPSS version 26.0 [17].

## Results

### Sociodemographic and health characteristics

A total of 201 participants were included in this study with a mean age of 39 ± 18 years. Most of the participants were female (56.2%), married (64.7%), living in urban areas (61.7%), had college degree or higher (56.7%), and had an annual household income of less than CNY 100,000 (58.8%). In addition, 32.3% of participants reported having chronic diseases, 68.2% reported fair sleep quality, 35.3% reported difficulties with walking, 14.4% reported self-care problems, and 63.7% reported pain or discomfort.

Among all participants, 69.2% reported having been diagnosed with a rare disease, while 42.8% had experienced misdiagnosis. Additionally, 54.2% had undergone genetic testing, 44.3% had received MDT services, and 79.6% had been hospitalized. Two-thirds of the participants had visited at least four hospitals for medical care. More than half of the participants perceived their current healthcare burden as very heavy, and more than one-third believed that the current treatments were effective. (Table 1)

### Psychological symptoms among participants

In this study, 68.2% of participants were identified as having depressive symptoms, including 38.8% (*n* = 78) with mild symptoms, 15.9% (*n* = 32) with moderate symptoms, and 13.4% (*n* = 27) with severe symptoms. Additionally, 41.3% (*n* = 83) of participants were identified as having anxiety symptoms, of whom 21.9% (*n* = 44) had mild symptoms, 12.4% (*n* = 25) had moderate symptoms, and 7% (*n* = 14) had severe symptoms. Notably, 37.8% (*n* = 76) of participants had both depressive and anxiety symptoms (Table 2).

### Factors associated with depressive symptoms and anxiety symptoms

Multivariable logistic regression analysis revealed that participants with poor sleep quality (aOR = 10.298, 95%CI: 1.915–55.367, compared to those with good sleep quality) and those experiencing pain or discomfort (aOR = 2.292, 95%CI: 1.143–4.596) were more likely to exhibit depressive symptoms. Higher social support (aOR = 0.893, 95%CI: 0.813–0.98) was associated with lower risk of experiencing depressive symptoms. Additionally, the coping styles of confrontation (aOR = 1.143, 95%CI: 1.042–1.253) and acceptance-resignation” (aOR = 1.166, 95%CI: 1.051–1.294) were positively associated with anxiety symptoms. Similarly, higher social support (aOR = 0.885, 95%CI: 0.817–0.959) was found to be a protective factor against anxiety symptoms (Table 3).

**Table 1:** Demographic characteristics of participants, stratified by psychological symptoms

Characteristics	Total	Depressive symptoms			Anxiety symptoms		
		No	Yes	χ/t	No	Yes	χ/t
**Age (years)**	39 ± 18	44 ± 17	43 ± 17	1.74*	43 ± 18	41 ± 16	0.927
**Gender**				0.827			1.569
Male	88 (43.8%)	31 (35.2%)	57 (64.8%)		56 (63.6%)	32 (36.4%)	
Female	113 (56.2%)	33 (29.2%)	80 (70.8%)		62 (54.9%)	51 (45.1%)	
**Marital status**				2.918*			0.009
Single and others	71 (35.3%)	28 (39.4%)	43 (60.6%)		42 (59.2%)	29 (40.8%)	
Married	130 (64.7%)	36 (27.7%)	94 (72.3%)		76 (58.5%)	54 (41.5%)	
**Residence (Hukou)**				1.949			1.535
Urban residence	124 (61.7%)	35 (28.2%)	89 (71.8%)		77 (62.1%)	47 (37.9%)	
Rural residence	77 (38.3%)	29 (37.7%)	48 (62.3%)		41 (53.2%)	36 (46.8%)	
**Educational level**				0.008			0.072
High school and below	87 (43.3%)	28 (32.2%)	59 (67.8%)		52 (59.8%)	35 (40.2%)	
College and above	114 (56.7%)	36 (31.6%)	78 (68.4%)		66 (57.9%)	48 (42.1%)	
**Annually family income (CNY)**				2.192			6.315*
< 5,0000	59 (29.4%)	16 (27.1%)	43 (72.9%)		29 (49.2%)	30 (50.8%)	
5,0001–10,0000	59 (29.4%)	21 (35.6%)	38 (64.4%)		38 (64.4%)	21 (35.6%)	
10,0001–15,000	36 (17.9%)	14 (38.9%)	22 (61.1%)		26 (72.2%)	10 (27.8%)	
> 15,0000	47 (23.3%)	13 (27.7%)	34 (72.3%)		25 (53.2%)	22 (46.8%)	
**History of chronic diseases**				1.432			4.389**
No	136 (67.7%)	47 (34.6%)	89 (65.4%)		45 (69.2%)	20 (30.8%)	
Yes	65 (32.3%)	17 (26.2%)	48 (73.8%)		73 (53.7%)	63 (46.3%)	
**Diagnosed with a rare disease**				1.504			1.111
No	62 (30.8%)	16 (25.8%)	46 (74.2%)		85 (61.2%)	54 (38.8%)	
Yes	139 (69.2%)	48 (34.5%)	91 (65.5%)		33 (53.2%)	29 (46.8%)	
**Have You Ever Been Misdiagnosed with Other Diseases?**				0.179			0.02
No	115 (57.2%)	38 (33%)	77 (67%)		50 (58.1%)	36 (41.9%)	
Yes	86 (42.8%)	26 (30.2%)	60 (69.8%)		68 (59.1%)	47 (40.9%)	
**Have You Ever Undergone Genetic Testing?**				0.155			0.081
No	92 (45.8%)	28 (30.4%)	64 (69.6%)		63 (57.8%)	46 (42.2%)	
Yes	109 (54.2%)	36 (33%)	73 (67%)		55 (59.8%)	37 (40.2%)	
**Received multidisciplinary team (MDT) services during treatment**				0.508			1.502
No	112 (55.7%)	38 (33.9%)	74 (66.1%)		48 (53.9%)	41 (46.1%)	
Yes	89 (44.3%)	26 (29.2%)	63 (70.8%)		70 (62.5%)	42 (37.5%)	
**History of hospitalizations**				0.145			0.145
No	41 (20.4%)	11 (26.8%)	30 (73.2%)		23 (56.1%)	18 (43.9%)	
Yes	160 (79.6%)	53 (33.1%)	107 (66.9%)		95 (59.4%)	65 (40.6%)	
**Number of hospitals visited**				2.988			0.989
1–3	71 (35.3%)	21 (29.6%)	50 (70.4%)		41 (57.7%)	30 (42.3%)	
4–6	78 (38.8%)	22 (28.2%)	56 (71.8%)		46 (59%)	32 (41%)	
7–9	29 (14.4%)	13 (44.8%)	16 (55.2%)		19 (65.5%)	10 (34.5%)	
10 and above	23 (11.5%)	8 (34.8%)	15 (65.2%)		12 (52.2%)	11 (47.8%)	
**Medical burden**				0.893			1.184
Heavy	100 (49.8%)	29 (29.0%)	71 (71.0%)		59 (59.0%)	41 (41.0%)	
Moderate	66 (32.8%)	22 (33.3%)	44 (66.7%)		36 (54.5%)	30 (45.5%)	
Light	35 (17.4%)	13 (37.1%)	22 (62.9%)		23 (65.7%)	12 (34.3%)	
**Effectiveness of current treatment**				4.982			3.807
Effective	74 (36.8%)	30 (40.5%)	44 (59.5%)		50 (67.6%)	24 (32.4%)	
Fair	58 (28.9%)	18 (31.0%)	40 (69.0%)		31 (53.4%)	27 (46.6%)	
Ineffective	34 (16.9%)	8 (23.5%)	26 (76.5%)		18 (52.9%)	16 (47.1%)	
Not Received Treatment	35 (17.4%)	8 (22.9%)	27 (77.1%)		19 (54.3%)	16 (45.7%)	
**Self-perceived burden**				4.602**			5.036**
No	94 (46.8%)	37 (39.4%)	57 (60.6%)		63 (67.0%)	31 (33.0%)	
Yes	107 (53.2%)	27 (25.2%)	80 (74.8%)		55 (51.4%)	52 (48.6%)	
**Sleep quality**				17.132**			5.478*
Good	30 (14.9%)	16 (53.3%)	14 (46.7%)		20 (66.7%)	10 (33.3%)	
Fair	137 (68.2%)	46 (33.6%)	91 (66.4%)		84 (61.3%)	53 (38.7%)	
Poor	34 (16.9%)	2 (5.9%)	32 (94.1%)		14 (41.2%)	20 (58.8%)	
**Experiencing problems with walking**				3.155*			0.483
No	130 (64.7%)	47 (36.2%)	83 (63.8%)		74 (56.9%)	56 (43.1%)	
Yes	71 (35.3%)	17 (23.9%)	54 (76.1%)		44 (62.0%)	27 (38.0%)	
**Experiencing problems with self-care**				0.927			1.521
No	172 (85.6%)	57 (33.1%)	115 (66.9%)		104 (60.5%)	68 (39.5%)	
Yes	29 (14.4%)	7 (24.1%)	22 (75.9%)		14 (48.3%)	15 (51.7%)	
**Experiencing pain or discomfort**				11.468**			4.529**
No	73 (36.3%)	34 (46.6%)	39 (53.4%)		50 (68.5%)	23 (31.5%)	
Yes	128 (63.7%)	30 (23.4%)	98 (76.6%)		68 (53.1%)	60 (46.9%)	
**Coping style**							
Confrontation	17.72 ± 4.2	17.51 ± 3.8	17.58 ± 4	0.346	16.98 ± 3.7	18.42 ± 4.2	2.569**
Avoidance	14.08 ± 3.2	15.01 ± 3.5	14.72 ± 3.4	1.816*	14.24 ± 3.4	15.4 ± 3.4	2.392**
Acceptance-resignation	7.81 ± 2.9	9.35 ± 3.5	8.86 ± 3.4	3.02**	7.98 ± 3.0	10.11 ± 3.6	4.531**
**Household annual income**	15.12 ± 16.9	13.51 ± 15	14.02 ± 15.6	0.68	15 ± 17.4	12.63 ± 12.6	1.058
**Social Support**	17.14 ± 3.4	14.8 ± 4.9	15.55 ± 4.6	3.918**	16.52 ± 4	14.17 ± 5.1	3.655**

*p < 0.1; **p < 0.05

**Table 2:** Prevalence of psychological symptoms among participants

Psychological symptoms	Total	Diagnosed patients	Undiagnosed patients
**Depressive symptoms (PHQ-9)**			
Minimal (0–4)	64 (31.9%)	48 (34.5%)	16 (25.8%)
Mild (5–9)	78 (38.8%)	56 (40.3%)	22 (35.5%)
Moderate (10–14)	32 (15.9%)	19 (13.7%)	13 (21%)
Severe (15–27)	27 (13.4%)	16 (11.5%)	11 (17.7%)
Positive for a depressive symptoms (PHQ-9 > 4)	137 (68.2%)	91 (65.5%)	46 (74.2%)
**Anxiety symptoms (GAD-7)**			
Minimal (0–5)	118 (58.7%)	85 (61.2%)	33 (53.2%)
Mild (6–9)	44 (21.9%)	30 (21.6%)	14 (22.6%)
Moderate (10–14)	25 (12.4%)	15 (10.8%)	10 (16.1%)
Severe (15–21)	14 (7.0%)	9 (6.5%)	5 (8.1%)
Positive for anxiety symptoms (GAD-7 > 5)	83 (41.3%)	54 (65.1%)	29 (34.9%)
**Comorbid anxiety and depressive symptoms**	76 (37.8%)	48 (34.5%)	28 (45.2%)

**Table 3:** The logistic regression analysis for influence factors of psychological symptoms

Psychological symptoms	Variables	OR (95%CI)	*P*
**Depressive symptoms**	**Age (year)**	1.01 (0.982–1.038)	0.491
	**Marital Status**		
	Single and others	Ref	
	Married	1.481 (0.577–3.801)	0.414
	**Self-perceived burden**		
	No	Ref	
	Yes	1.539 (0.766–3.093)	0.226
	**Sleep Quality**		
	Good	Ref	
	Fair	1.591 (0.647–3.911)	0.312
	Poor	10.298 (1.915–55.367)	**0.007**
	**Experiencing pain or discomfort**		
	No	Ref	
	Yes	2.292 (1.143–4.596)	**0.019**
	**Experiencing problems with walking**		
	No	Ref	
	Yes	1.024 (0.465–2.253)	0.954
	**Coping style**		
	Avoidance	1.09 (0.98–1.213)	0.112
	Acceptance-resignation	1.117 (0.998–1.251)	0.054
	**Social Support**	0.893 (0.813–0.98)	**0.017**
**Anxiety symptoms**	**Annually family income (CNY)**		
	< 5,0000	Ref	
	5,0001–10,0000	0.724 (0.308-1.700)	0.459
	10,0001–15,000	0.475 (0.167–1.355)	0.164
	> 15,0000	0.951 (0.388–2.334)	0.913
	**History of Chronic Diseases**		
	No	Ref	
	Yes	0.596 (0.288–1.233)	0.163
	**Self-perceived burden**		
	No	Ref	
	Yes	1.366 (0.688–2.712)	0.373
	**Sleep Quality**		
	Good	Ref	
	Fair	0.844 (0.326–2.182)	0.726
	Poor	1.408 (0.438–4.523)	0.565
	**Experiencing pain or discomfort**		
	No	Ref	
	Yes	1.76 (0.879–3.525)	0.111
	**Coping style**		
	Confrontation	1.143 (1.042–1.253)	**0.004**
	Avoidance	1.083 (0.979–1.198)	0.123
	Acceptance-resignation	1.166 (1.051–1.294)	**0.004**
	**Social Support**	0.885 (0.817–0.959)	**0.003**

## Discussion

This study explored the mental health status of patients consulting a rare disease outpatient clinic for rare and undiagnosed disease in China and identified associated factors. We found a high prevalence of depressive and anxiety symptoms among these patients, with more than one-third experiencing both conditions. These findings indicate a substantial mental health burden in this population and highlight the importance of integrating psychological assessment and support into multidisciplinary rare disease care.

This study highlights a markedly increased risk of depression in patients with rare diseases experiencing poor sleep quality. Notably, studies report a 27% prevalence of depressive symptoms among outpatients, significantly surpassing that of healthy populations [18]. However, the sleep quality and depression prevalence in rare disease patients remain largely underestimated. This underestimation may stem from diagnostic delays, difficulties in managing chronic symptoms, and the considerable psychological stress endured by these patients. Compared to the general population, the interplay between sleep and depression in rare disease patients exhibits distinctive features that warrant closer examination. On one hand, psychoneuroimmunological connections between depressive symptoms and medical conditions are well-documented, with the pathophysiology of rare diseases potentially disrupting sleep regulation mechanisms [19]. This connection is often mediated by inflammatory mechanisms. Research shows that inflammatory markers, such as C-reactive protein and interleukins, are elevated in depressive patients and serve as predictors of depressive onset [20]. Rare disease patients, frequently presenting chronic inflammatory traits such as those seen in autoinflammation or metabolic conditions, further amplify the influence of the inflammation-depression pathway. Experimental activation of inflammatory responses not only exacerbates depressive symptoms but also stimulates brain regions responsible for regulating positive and negative emotions [21, 22]. In rare disease patients, such neural responses are likely intensified by the compounded stress of the illness and prolonged diagnostic delays. Sleep disturbances diminish the ability to recover positive emotions, leaving rare disease patients more vulnerable to feelings of helplessness and despair when confronting their condition [23]. Emerging evidence suggests that insomnia is not merely a secondary symptom of depression but an independent risk factor, offering a prospective signal for depressive onset and a vital early warning for clinical intervention [24–26]. Consequently, integrating routine sleep screening, particularly for patients with persistent insomnia, into rare disease management is crucial. Effective interventions, such as cognitive behavioral therapy for insomnia or melatonin-based pharmacotherapy, may significantly enhance sleep quality and reduce depression risk [27].

Persistent pain or discomfort significantly heightens the risk of depression in rare disease patients, particularly among those with unresolved chronic pain. The high comorbidity of pain and depression in rare disease patients underscores the intricate interplay between physical and psychological burdens. Chronic pain, as a stressor, is a pivotal determinant of depression, and their coexistence often exacerbates both conditions [28]. Pain and stress exposure activates the HPA axis, disrupting neurotransmitter balance (e.g., 5-HT and dopamine), thereby intensifying depressive symptoms [29–32]. Additionally, pain diminishes patients’ sense of control, triggering helplessness and social withdrawal, which further amplifies psychological stress. Therefore, an integrated approach combining pharmacological and psychological interventions is crucial for pain management in rare disease patients. At the therapeutic level, the limited tolerance and complex drug interactions in rare disease patients restrict the use of conventional treatments for sleep or depression. Clinicians must balance the risks and benefits, addressing both the primary disease and associated sleep and depression issues, highlighting the unique challenges in managing these interconnected conditions.

Social support plays a crucial protective role in the mental health of rare disease patients. High levels of social support significantly alleviate depressive symptoms. It fosters emotional belonging and security, reducing anxiety and depression stemming from prolonged disease uncertainty [33]. By lowering stress hormone levels, social support may indirectly enhance sleep quality and pain perception, alleviating psychological burdens [34]. Policy-driven initiatives, such as patient support groups and volunteer programs, alongside strengthened family and social networks, are essential. From a macro-policy perspective, rare diseases have emerged as a critical focus in China’s policy landscape. The current window of opportunity necessitates the establishment of a robust rare disease support system centered on a foundational legal framework. This should include policies for medical and social services, research, collaboration, funding, and oversight to enhance societal awareness and investment [35]. Efforts should also target reducing systemic inefficiencies, such as duplicative forms, complex instructions, and prolonged waiting times, to streamline the patient journey [36].

Additionally, a complex interplay exists among sleep, pain, and social support in rare disease patients: sleep disturbances heighten pain sensitivity, while chronic pain further impairs sleep quality. This vicious cycle is magnified by the prolonged symptom burden in rare diseases, potentially forming a central pathological basis for depression [37, 38]. Social support acts as a moderator, alleviating psychological stress and improving sleep and pain experiences, thereby mitigating the negative impact on depression. Future studies should develop an interactive model linking sleep, pain, depression, and social support, using longitudinal research to clarify causal relationships and inform multidimensional intervention strategies.

## Limitations

Several limitations should be considered when interpreting the findings of this study. First, as an inherent constraint of the real-world clinical setting, this study grouped all participants under the broad term of ‘rare diseases’ without stratifying them by specific disease types, genetic etiologies, or clinical subgroups. Consequently, this lack of stratification limits our ability to detect disease-specific variations in psychological distress. Second, the single-center and cross-sectional design of this study presents certain constraints. The cross-sectional nature precludes causal inferences, while reliance on a single center may limit the generalizability of our findings. Therefore, future multicenter, longitudinal studies are required to validate these results in broader populations. Third, the potential for somatic confounding in our questionnaire assessment must be acknowledged. We utilized the PHQ-9 and GAD-7, which inherently include somatic items such as sleep disturbances, fatigue, and appetite changes. Because rare diseases impose a severe physical burden, these physiological manifestations may overlap with symptoms of psychological distress, potentially inflating the total scores. Consequently, the elevated depression and anxiety levels observed in our study likely reflect a compounded biopsychosocial burden rather than a purely affective mood disorder alone.

## Conclusion

In this study, 68.2% of patients with rare and undiagnosed diseases were identified as having depressive symptoms. Effective interventions, including sleep screening, cognitive behavioral therapy, and pain management, are essential. Future research should explore the interconnectedness of these factors to develop comprehensive treatment strategies for rare disease patients.

## Data Availability

The datasets generated and analyzed during the current study are not publicly available due to privacy concerns regarding identifiable patient information but are available from the corresponding author on reasonable request.
